# Osteosarcoma with cell-cycle and fibroblast growth factor genomic alterations: case report of Molecular Tumor Board combination strategy resulting in long-term exceptional response

**DOI:** 10.1186/s13045-022-01344-x

**Published:** 2022-08-28

**Authors:** Hanna E. Persha, Shumei Kato, Pradip De, Jacob J. Adashek, Jason K. Sicklick, Vivek Subbiah, Razelle Kurzrock

**Affiliations:** 1 College of Pharmacy, Purdue University, West Lafayette, IN USA; 2 Center for Personalized Cancer Therapy and Division of Hematology and Oncology, Department of Medicine, UC San Diego Moores Cancer Center, 3855 Health Sciences Drive, La Jolla, CA 92093 USA; 3 Avera Cancer Institute, Sioux Falls, SD USA; 4 Department of Oncology, The Sidney Kimmel Comprehensive Cancer Center, The Johns Hopkins Hospital, Baltimore, MD USA; 5 Division of Surgical Oncology, Department of Surgery, Center for Personalized Cancer Therapy, University of California San Diego, La Jolla, CA USA; 6 Division of Cancer Medicine, Department of Investigational Cancer Therapeutics (Phase 1 Clinical Trials Program), University of Texas MD Anderson Cancer Center, Houston, TX USA; 7 Genomic Sciences and Precision Medicine Center, Medical College of Wisconsin, Milwaukee, WI USA; 8 WIN Consortium, Paris, France

**Keywords:** Osteosarcoma, Targeted therapy, Precision, Genomic

## Abstract

There is a paucity of information about molecularly driven therapy in osteosarcomas. We report a 31-year-old woman with chemotherapy–refractory metastatic osteosarcoma who was successfully treated with the combination of palbociclib (CDK4/6 inhibitor) and lenvatinib (multikinase FGFR inhibitor), selected based on next generation sequencing that showed *CDK4* and *CCND2* amplifications (upregulates CDK4/6), and *FGF6* (ligand for FGFR1,2 and 4), *FGF23* (ligand for FGFR1,2,3, and 4) and *FRS2* (adaptor protein for FGFR signaling) amplifications. The patient’s tumor showed 68% reduction in positron emission tomography (PET) avidity, lasting 31 months after therapy initiation, when a solitary recurrence occurred, was resected, and treatment continued. The patient remains on matched targeted therapy at 51 + months from the start of the combination. Treatment was given at reduced dosing (lenvatinib 10 mg oral daily (approved dose = 24 mg daily)) and palbociclib 75 mg oral daily, one week on and one week off (approved dose = 125 mg oral daily, three weeks on/one week off) and is tolerated well. Therefore, co-targeting the aberrant cyclin and FGFR pathways resulted in long-term exceptional response in a patient with refractory advanced osteosarcoma.

**To the Editor,**

Osteosarcoma is the most common primary bone tumor in children and young adults. Unfortunately, metastatic disease treated with front-line chemotherapy shows a three-year event-free survival of only 32% [1].

At the molecular level, osteosarcomas most commonly harbor alterations in *TP53* (74%), *RB1* (64%), and *MYC* (39%), which are challenging targets [2]. However, some osteosarcomas have potentially actionable targets, including *CDK4* (11%) and *PTEN* (56%) alterations [2].

A limitation of targeted therapy in osteosarcoma and other malignancies could be due to multiple co-existing driver molecular alterations in metastatic disease [3–5]. For instance, amongst 31 osteosarcomas, there was a median of 21 single-nucleotide variants/cancer (whole exome sequencing) [6]. Similarly, in 112 osteosarcomas who underwent exome or whole genome sequencing, another report found a median of 38 mutations per tumor. Even so, clinical trials that utilize genomic biomarkers generally only target one gene at a time. However, recent data suggests that customized combinations of drugs matched to genomic alterations can be safe and effective across cancer types [4, 5, 7, 8].

Herein, we describe a patient with treatment-refractory metastatic osteosarcoma who was successfully managed long-term with a genomically matched (chemotherapy-free) combination strategy.

## Case presentation

A 30-year-old woman with recurrent, refractory metastatic osteosarcoma was referred. At age 17, she underwent limb-sparing surgery for a left leg (femur) periosteal osteosarcoma. Eleven years later, computerized tomography (CT) detected a right hilar mass and multiple pulmonary nodules. Lung lesion resection confirmed osteosarcoma, and was followed by adriamycin and cisplatin and later adriamycin and ifosfamide administration (total = 6 cycles). Eight months later, CT scan revealed new pulmonary lesions. She received ifosfamide (progression-free survival [PFS] = 4 months) followed by stereotactic body radiation therapy and ipilimumab (clinical trial) (PFS = 21 months). Tissue was then analyzed by next-generation sequencing (Foundation Medicine (https://www.foundationmedicine.com/) (*N* = 405 genes)), which revealed *CDK4*, *MDM2* and *FRS2* amplification (≥ 8 copy number alterations) as well as 6–7 copy number amplifications in *CCND2*, *FGF6* and *FGF23.* The patient was referred to the University of California San Diego (UCSD) Moores Cancer Center Molecular Tumor Board (MTB).

**Fig. 1:**
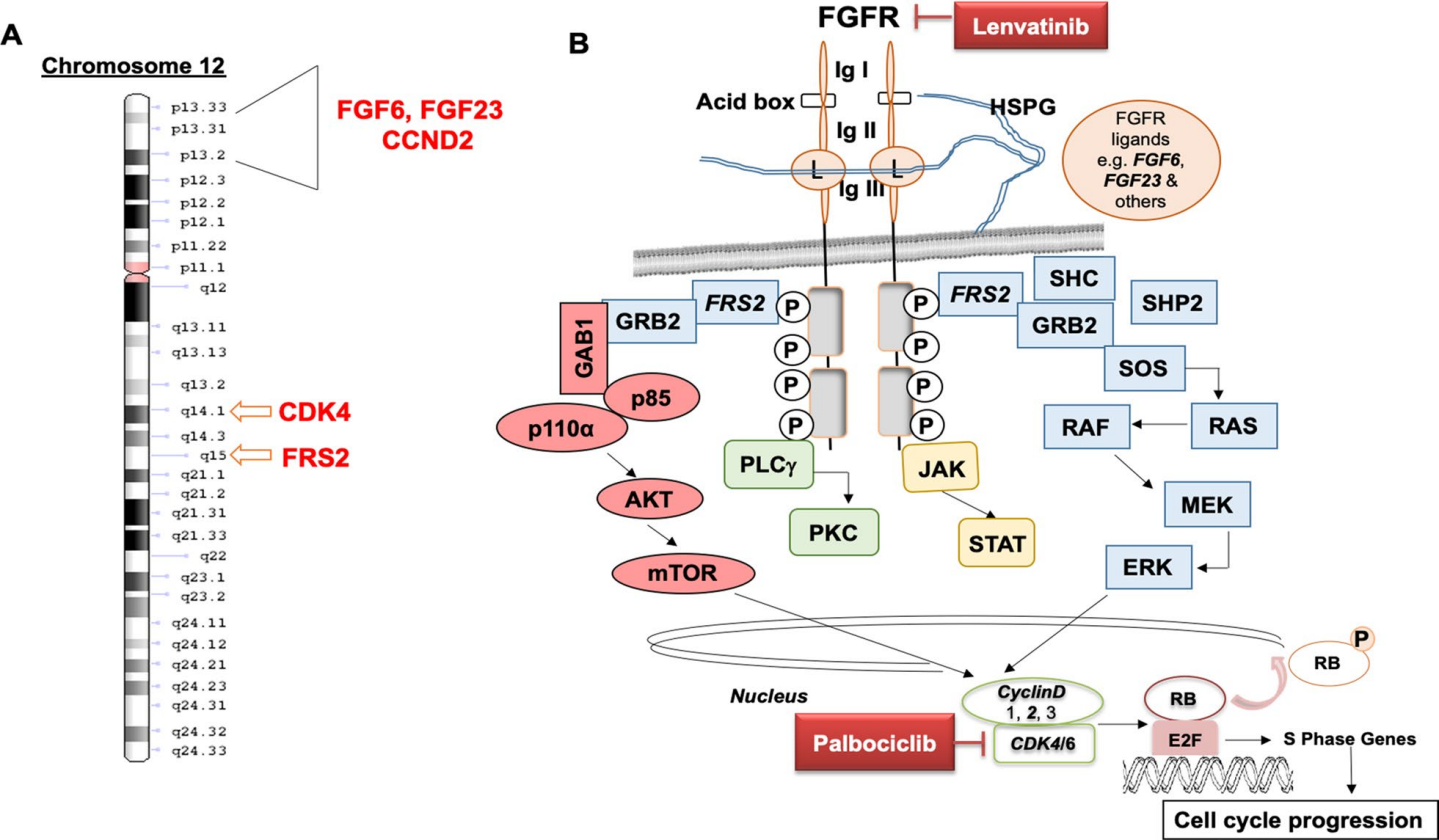
Chromosomal localization of the patient’s amplified genes *CDK4*, *MDM2*, *FRS2*, *CCND2*, *FGF6* and *FGF23* and FGF-FGFR signaling pathways cross talk with cell-cycle pathway. **A** Relevant (targeted) amplified genes are detected in chromosome 12, and their specific localization are demonstrated in the figure. MDM2 was also amplified and localizes to chromosome 12, but it was not considered druggable. **B** The binding of ligands to receptors triggers the conformational changes of FGFRs, leading to dimerization and activation of FGFRs. Activated FGFRs phosphorylate FRS2, and FRS2 binds to the SH2 domain-containing adaptor protein GRB2. GRB2 will subsequently bind to SHC, SOS and activates downstream the RAS-RAF-MEK-ERK pathway responsible for proliferation and survival. GRB2 also binds with another adaptor protein, GAB1, which has a YXXM motif responsible for the recruitment of p85, leading to activate the PI3K-AKT-mTOR pathway. The PI3K-AKT-mTOR pathway is responsible for proliferation, migration, angiogenesis, cap-dependent mRNA translation and inhibits apoptosis. Cyclin D1/D2/D3 is also activated by upstream RAS-MAPK and AKT-mTOR pathways. Cyclin D binds with CDK4/6 to promote RB phosphorylation, which depresses the E2F transcription factor to drive the expression of genes that promote cell cycle progression. The FGF-FGFR signaling pathway also activates downstream JAK-STAT and PLCγ-PKC pathways, both are responsible for various oncogenic phenotypes. Amplified genes from current case (*FGF6, 23, FRS2, Cyclin D2, and CDK4*) are showing in italic, and therapies (palbociclib and lenvatinib) are showing in the red boxes. *FGF* fibroblast growth factor, *FGFR* fibroblast growth factor receptor, *FRS2* FGFR substrate 2, *GAB1* GRB2 associated binding protein 1, *GRB2* growth factor receptor-bound 2, *SOS* son of sevenless; *PKC* protein kinase C, *PLCγ* phospholipase C gamma, *HSPG* heparan sulfate proteoglycan

**Fig. 2:**
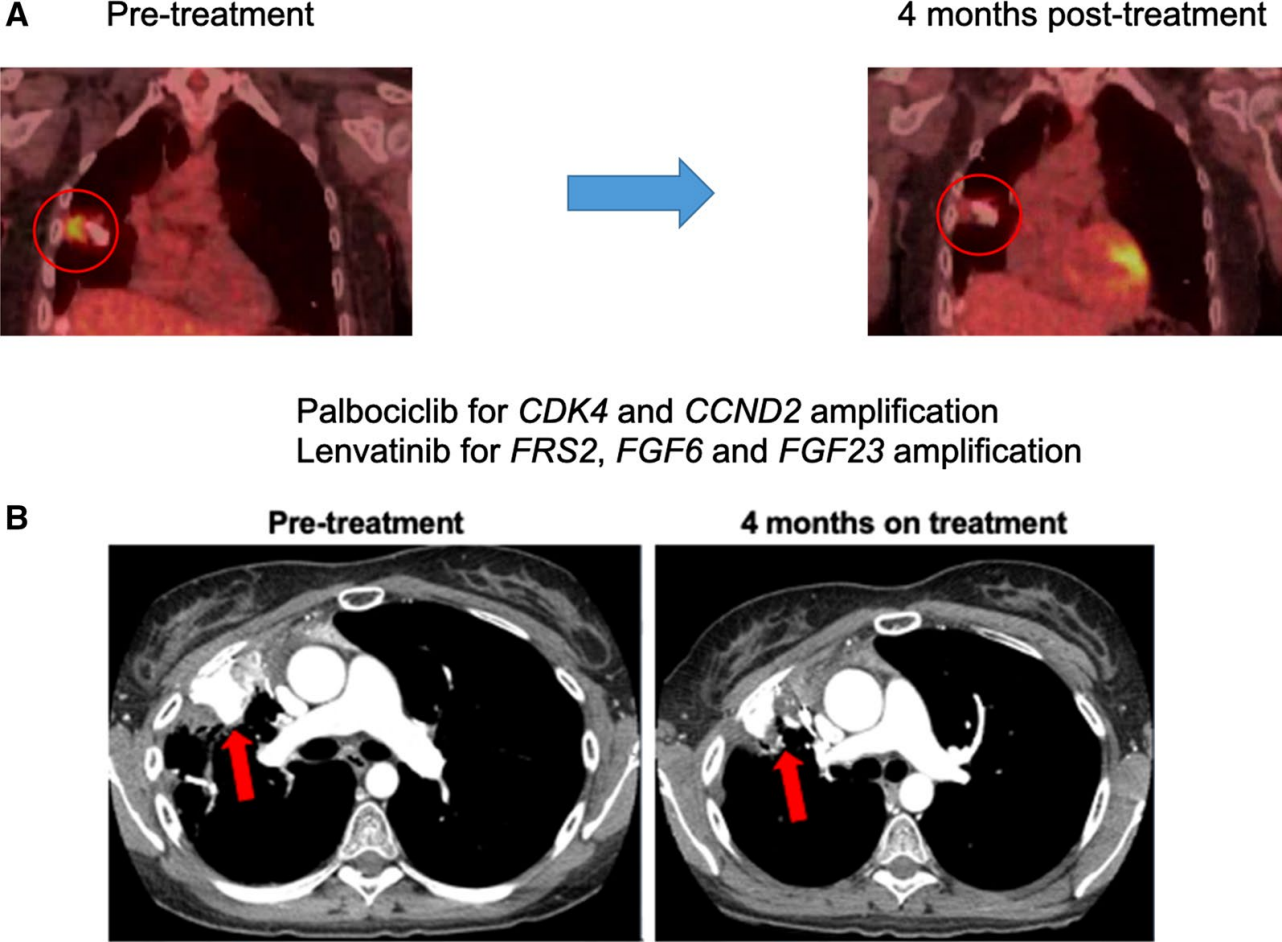
Osteosarcoma patient with multiple recurrences whose tumor progressed on several lines of therapy, now treated successfully with matched targeted combination treatment approach. Therapy ongoing at 51 + months. **A** (PET scan) and **B** (CT scan). Osteosarcoma patient with multiple recurrences whose tumor progressed on several lines of therapy, now treated successfully with matched targeted combination treatment approach. Therapy ongoing at 51 + months

The UCSD MTB is a tumor-agnostic tumor board comprised of medical, surgical and radiation oncologists, radiologists and pathologists, bioinformatics specialists and basic scientists, clinical study coordinators and navigators, and medication acquisition specialists [4] that focuses on discussing therapies based on patients’ tumor multi-omic results. The MTB recommended combination therapy with palbociclib (CDK4/6 inhibitor for *CDK4* and *CCND2* amplifications) and lenvatinib (an FGFR inhibitor for *FGF6* (ligand for FGFR1,2 and 4), *FGF23* (ligand for FGFR1,2,3, and 4) and *FRS2* (adaptor protein for FGFR signaling) amplifications) (Fig. 1) (50% inhibitory concentration (IC50) for CDK4 with palbociclib: 9 nM; IC50 for FGFR1-4 with lenvatinib: 27–61 nM (IC50 was determined from FDA pharmacological reviews (available online))). The patient signed consent for the PREDICT study (NCT02478931). She began palbociclib 75 mg orally/day (three weeks on/one week off) and lenvatinib 10 mg orally/day. Thrombocytopenia necessitated dose reduction of palbociclib to 75 mg/day, one week on/one week off (approved palbociclib dose = 125 mg orally/day, 3 weeks on/one week off). Lenvatinib was increased to 14 mg orally/day; however, due to mucositis, the dose was re-reduced to 10 mg orally/day (approved lenvatinib dose = 24 mg orally/day). Four months later, positron emission tomography (PET)/computed tomography (CT) scan demonstrated marked improvement in mid-right lung mass PET avidity (Fig. 2A) while a CT scan (Fig. 2B) showed overall stable disease (68% reduction in PET avidity: SUV = 4 [before therapy] down to SUV = 1.3 [nadir]). Subsequent images showed no evidence of progression until 31 months later, when a right hemi-diaphragm mass appeared. The mass was resected and confirmed to be osteosarcoma. Molecular profiling on the cartilaginous surgical sample failed. Post-surgery, the patient resumed palbociclib and lenvatinib. Therapy has been ongoing for 51 + months since its initiation, with excellent tolerance and continued response.

## Discussion

Relapsed/refractory osteosarcoma is a challenging disease. Periosteal osteosarcoma is very rare and has a better prognosis than conventional osteosarcoma. However, this patient had metastatic disease that had progressed after several chemotherapies. There is high molecular diversity in advanced osteosarcoma with several undruggable (to date) targets (such as TP53 and Rb). To date, precision therapies and direct bone targeting therapies such as alpha particle radium 223 have demonstrated limited activity [9]. Moreover, genomically matched CDK4/6 inhibitors (e.g., palbociclib, ribociclib and abemaciclib) as well as matched FGFR inhibitors as monotherapy have shown limited responses across malignancies [10–12]. CDK4/6 inhibitors have been used in liposarcoma, with activity. However, there are no trials published of CDK4/6 inhibitors in osteosarcoma; a single case report showed stable disease for about 10 months in a patient give the CDK4/6 inhibitor, ribociclib, together with gemcitabine.

In this context, the importance of the current case lies in showcasing the activity of molecularly matched combination therapy, specifically with a CDK4/6 inhibitor given together with a multi-kinase FGFR inhibitor for a relapsed osteosarcoma. Of note, not only were the drugs matched to the patient’s genomic alterations, but the dose of each drug was reduced from the approved dose and hence tailored to the patient’s tolerance. Most remarkably, the patient is doing well on the palbociclib with lenvatinib combination at over four years—51 + months. Further studies co-targeting FGFR and CDK4/6 signals in patients whose tumors harbor cognate alterations are warranted.

## Data Availability

N/A.
